# Reverse arthroplasty compared to hemiarthroplasty and open reduction and internal fixation for displaced proximal humerus fracture in patients above 60: a Bayesian network meta-analysis

**DOI:** 10.1007/s00402-025-06067-5

**Published:** 2025-10-18

**Authors:** Filippo Migliorini, Giuliano Sammaria, Luise Schäfer, Michael Memminger, Francesco Simeone, Nicola Maffulli

**Affiliations:** 1 Department of Trauma and Reconstructive Surgery, University Hospital in Halle, Martin-Luther University Halle-Wittenberg, 06097 Halle (Saale), Germany; 2 Department of Orthopaedic and Trauma Surgery, Academic Hospital of Bolzano (SABES-ASDAA), via Lorenz Böhler 5, 39100 Bolzano, Italy; 3 Department of Life Sciences, Health, and Health Professions, Link Campus University, Via delCasale di San Pio V, 00165 Rome, Italy; 4 University of Salerno, Fisciano, Italy; 5 Department of Orthopaedic and Trauma Surgery, Eifelklinik St.Brigida, Kammerbruschstr. 8, 52152 Simmerath, Germany; 6 Faculty of Medicine and Psychology, University “La Sapienza” of Rome, Rome, Italy; 7 School of Pharmacy and Bioengineering, Keele University Faculty of Medicine, Stoke on Trent, ST4 7QB London, United Kingdom; 8 Centre for Sports and Exercise Medicine,Queen Mary University of London, Barts and the London School of Medicine and Dentistry, MileEnd Hospital, 275 Bancroft Road, E1 4DG London, United Kingdom

**Keywords:** Displaced shoulder fracture, Arthroplasty, Hemiarthroplasty, Reverse total shoulder arthroplasty, rTSA, Open reduction and internal fixation, ORIF, Elderlies, Aged

## Abstract

**Introduction:**

The present Bayesian network meta-analysis compared reverse total shoulder arthroplasty (rTSA) to hemiarthroplasty (HA) and open reduction and internal fixation (ORIF) for displaced proximal humeral fractures (PHF) in patients above 60.

**Methods:**

This study was conducted according to the PRISMA extension statement for reporting systematic reviews incorporating network meta-analyses of healthcare interventions. In December 2024, PubMed, Web of Science, and Embase databases were accessed. No time constraint was set for the search. All clinical studies were accessed comparing rTSA, ORIF and HA for displaced PHF in patients older than 60. Only studies which compared at least two of the interventions of interest were eligible when they reported a minimum of 12 months of follow-up. Two-, three-, and four-part displaced fractures and fractures with a head-splitting component were considered.

**Results:**

Data from 878 procedures were collected. 72% (631 of 878 patients) were women. The mean length of the follow-up was 33.8 ± 14.8 months. The mean age of the patients was 73.4 ± 4.6 years. Between groups, comparability was found in the mean age, the ratio of men to women, the length of the follow-up, and the time elapsed from injury to the procedure. The rTSA demonstrated the lowest rate of complications, followed by HA and ORIF. The rTSA demonstrated the lowest rate of revision, followed by HA and ORIF. Given the limited and heterogeneous data, only complications and revision rates were analysed in the network meta-analysis; functional outcomes were discussed narratively.

**Conclusion:**

In patients above 60 with displaced PHF, rTSA was associated with a lower complication and revision rate than ORIF and HA.

**Level of evidence:**

Level IV, systematic review and meta-analysis.

**Supplementary Information:**

The online version contains supplementary material available at 10.1007/s00402-025-06067-5.

## Introduction

Proximal humeral fractures (PHF) account for up to 6% of all adult fractures, representing the third most common type of fracture, particularly in older women [1–7]. Additional risk factors are diabetes, epilepsy, depression, and dementia [8]. Despite PHF being common [9, 10], the most appropriate treatment remains controversial [11, 12], with some level I investigations recommending conservative management of three- and four-part fractures [13, 14]. Age, bone quality, comorbidities, instability, general condition of the patient [15–20], and the type of fracture are the most important prognostic factors [21, 22]. In patients not suitable for surgery and minimally displaced or hairline fractures, a nonoperative approach is advocated [23–26]. Surgery is, however, still performed for displaced and comminuted fractures [21, 27]. Open reduction and internal fixation (ORIF), hemiarthroplasty (HA), and reverse total shoulder arthroplasty (rTSA) are the most common surgical procedures for PHF in older adults [28–30].

However, in elderly patients with displaced PHF, the choice among ORIF, HA, and rTSA remains clinically debated. ORIF is typically preferred in less comminuted fractures where anatomical reduction is feasible, although outcomes may be compromised by osteoporotic bone and the risk of avascular necrosis [31–35]. HA was introduced to address these limitations, but it depends on tuberosity healing and rotator cuff integrity, which are often impaired in older patients [28, 36]. More recently, rTSA has emerged as a reliable option that bypasses these anatomical constraints, offering predictable functional outcomes even without tuberosity repair [2, 37–39]. However, questions remain regarding its precise indications and long-term results [40, 41]. These treatment considerations underscore the need for a comprehensive comparison across all three modalities in this patient population.

Previous meta-analyses have provided valuable insights into selected comparisons among rTSA, HA, and ORIF. However, a comprehensive synthesis encompassing all three modalities remains lacking [42, 43]. In addition, several studies have been published since the most recent review by Suroto et al. [43], reinforcing the need for an updated and methodologically advanced appraisal of the literature. Accordingly, a Bayesian network meta-analysis was conducted to compare ORIF, HA, and rTSA for treating displaced PHF in older adults, integrating direct and indirect evidence. By focusing on complication and revision rates, this study aims to support contemporary clinical decision-making in the surgical management of PHF. The authors hypothesised that rTSA would result in lower complication and revision rates than ORIF and HA.

## Methods

### Eligibility criteria

All the clinical studies published in peer-reviewed journals investigating rTSA, ORIF, and HA in the management of PHF were accessed. Only studies which compared at least two of the interventions of interest were eligible. Only studies that reported a minimum of 12 months of follow-up were considered. Studies that combined two or more interventions were not considered, nor were those which reported data on experimental pre- or post-operative procedures. Only studies which included patients with a minimum age of 60 years were eligible. According to the author´s language capabilities, English, Italian, German, Spanish, and French articles were eligible. Only studies with levels I–IV of evidence, according to the Oxford Centre of Evidence-Based Medicine [44], were considered. Reviews, opinions, letters, and editorials were not considered. Animals, in vitro, biomechanics, computational, and cadaveric studies were not eligible. Missing quantitative data on outcomes of interest warranted the exclusion of the study.

### Search strategy

This study was conducted according to the Preferred Reporting Items for Systematic Reviews and Meta-Analyses: the 2020 PRISMA statement [45]. The PICOTD algorithm was preliminarily established:

- P (Problem): PHF in patients above 60;
- I (Intervention): surgical management;
- C (Comparison): rTSA, ORIF, HA;
- O (Outcomes): revisions and complications;
- T (Timing): minimum 12 months follow-up;
- D (Design): clinical study.

In December 2024, the following databases were accessed: PubMed, Web of Science, and Embase. No time constraint was set for the search. The Medical Subject Headings (MeSH) used for the database search are reported in Appendix A. No additional filters were used in the database search.

### Selection and data collection

Two authors (**;**) independently performed the database search. All the resulting titles were screened by hand, and the abstract was accessed if suitable. The full text of the abstracts that matched the topic of interest was accessed. If the full text was not accessible or available, the article was not considered for inclusion. A cross-reference search of the bibliography of the full-text articles was also performed to identify additional relevant articles. Disagreements were debated and mutually solved by the authors. A third senior author (**) made the final decision in case of further disagreement.

### Data items

Two authors (**;**) independently performed data extraction. The following data at baseline were extracted: author, year of publication, journal, length of follow-up, number of patients, mean age of patients with related conditions, and BMI. Data concerning complications and revisions were collected. Elective hardware removal was not considered a revision or complication. Data were extracted to Microsoft Office Excel version 16.72 (Microsoft Corporation, Redmond, USA).

### Methodological quality assessment and quality of the recommendations

The risk of bias was evaluated following the guidelines in the Cochrane Handbook for Systematic Reviews of Interventions [46]. Two reviewers (**;**) independently assessed the risk of bias in the extracted studies. Disagreements were solved by a third senior author (**). Nonrandomised controlled trials (non-RCTs) were evaluated using the Risk of Bias in Nonrandomised Studies of Interventions (ROBINS-I) tool [47]. Seven domains of potential bias in non-RCTs were assessed. Possible cofounding and the nature of patient selection before the start of the comparative intervention are assessed by two domains. A further domain assesses bias in the classification during the intervention. The final four domains assess the methodological quality after the intervention comparison has been implemented and relate to deviations from previously intended interventions, missing data, erroneous measurement of outcomes, and bias in the selection of reported outcomes. The figure of the ROBINS-I was elaborated using the Robvis Software (Risk-Of-Bias VISualization, Riskofbias.info, Bristol, UK) [48].

### Synthesis methods

The main author (**) performed the statistical analyses following the recommendations of the Cochrane Handbook for Systematic Reviews of Interventions [49]. Baseline demographics were assessed through the IBM SPSS (International Business Machines Corporation, Armonk, USA) software. Mean and standard deviation were used for continuous variables, and frequency (events/ observations) for binary endpoints. Analysis of variance (ANOVA) was used to assess baseline comparability, with values of P > 0.1 considered satisfactory. The network meta-analyses were conducted using the STATA Software/MP, version 14.1 (StataCorporation, College Station, Texas, USA). The STATA routine for Bayesian hierarchical random-effects model analysis was used. The Log Odd Ratio (LOR) effect measure was adopted to analyse dichotomic data. The analyses of complications and revisions were conducted in a Bayesian hierarchical framework, which accommodates treatment arms with zero events without the need for continuity correction. The overall inconsistency was evaluated through the equation for global linearity via the Wald test. If P_Wald_ > 0.5, the null hypothesis could not be rejected, and the consistency assumption could be accepted at the overall level of each treatment. Both confidence (CI) and percentile (PrI) intervals were 95%. Edge and interval plots were obtained and evaluated. The funnel plot of each comparison was performed to assess data dispersion. The funnel plot of the most commonly reported outcome was used to determine the risk of publication bias. Egger’s linear regression was performed to assess asymmetry in the funnel plot, with values of P_Egger_ < 0.05 indicating statistically significant asymmetry.

## Results

### Study selection

**Fig. 1:**
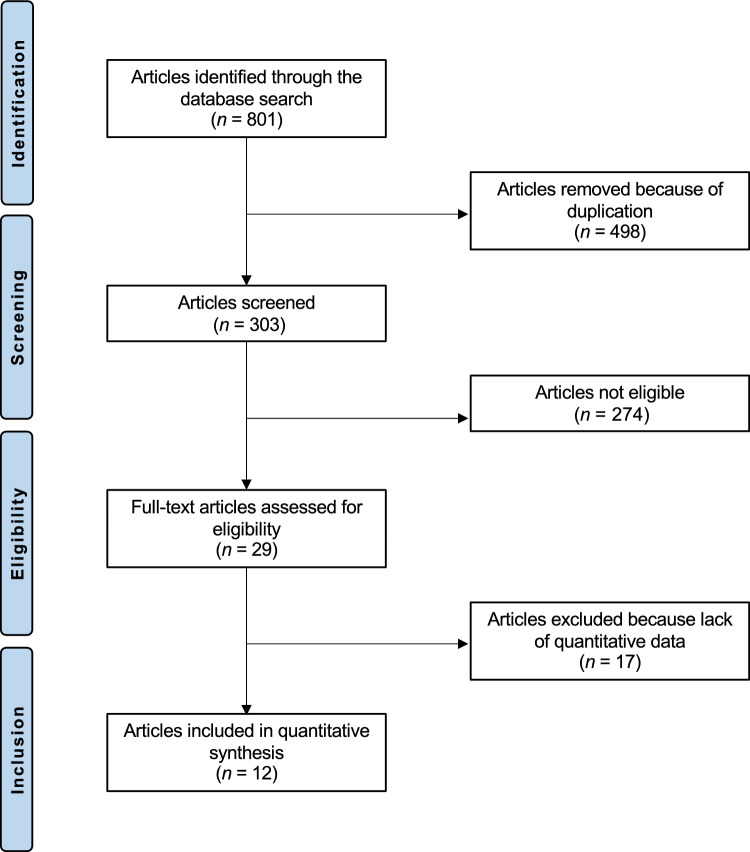
PRISMA flow chart of the literature search

The systematic literature search revealed a total of 801 studies. Of them, 498 were identified as duplicates and therefore excluded. Screening the abstracts of the remaining 303 investigations for eligibility resulted in excluding an additional 274 articles. The detailed reasons that led to exclusion were the following: study type and design (*N* = 113), low level of evidence (*N* = 36), not comparing at least two of the interventions of interest (rTSA and ORIF and HA) (*N* = 43), combining two or more of the interventions (*N* = 22), reporting data on experimental pre- or post-operative procedures (*N* = 17), patients studied aged less than 60 years (*N* = 21), follow-up shorter than 12 months (*N* = 16), and language limitations (*N* = 6). A further 17 studies missed quantitative data under the outcomes of interest and were not considered. In conclusion, 12 clinical trials were selected for inclusion in the present investigation. The study design was prospective in three studies, and nine had a retrospective study design. The results of the literature search are shown in Fig. 1.

### Risk of publication bias

**Fig. 2:**
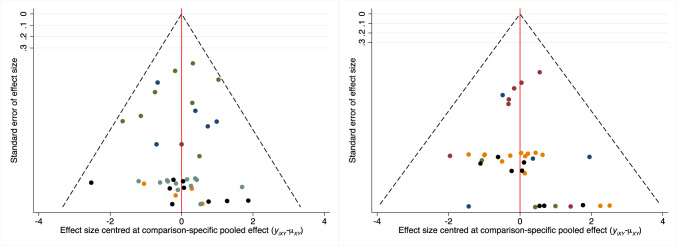
Funnel plot endpoint complications (*left*) and revisions (*right*)

Egger’s test of the endpoint complications (P_Egger_ = 0.5) and revisions (P_Egger_ = 0.6) found no statistically significant asymmetry, indicating a low risk of bias at the overall publication level (Fig. 2).

### Methodological quality assessment

**Fig. 3:**
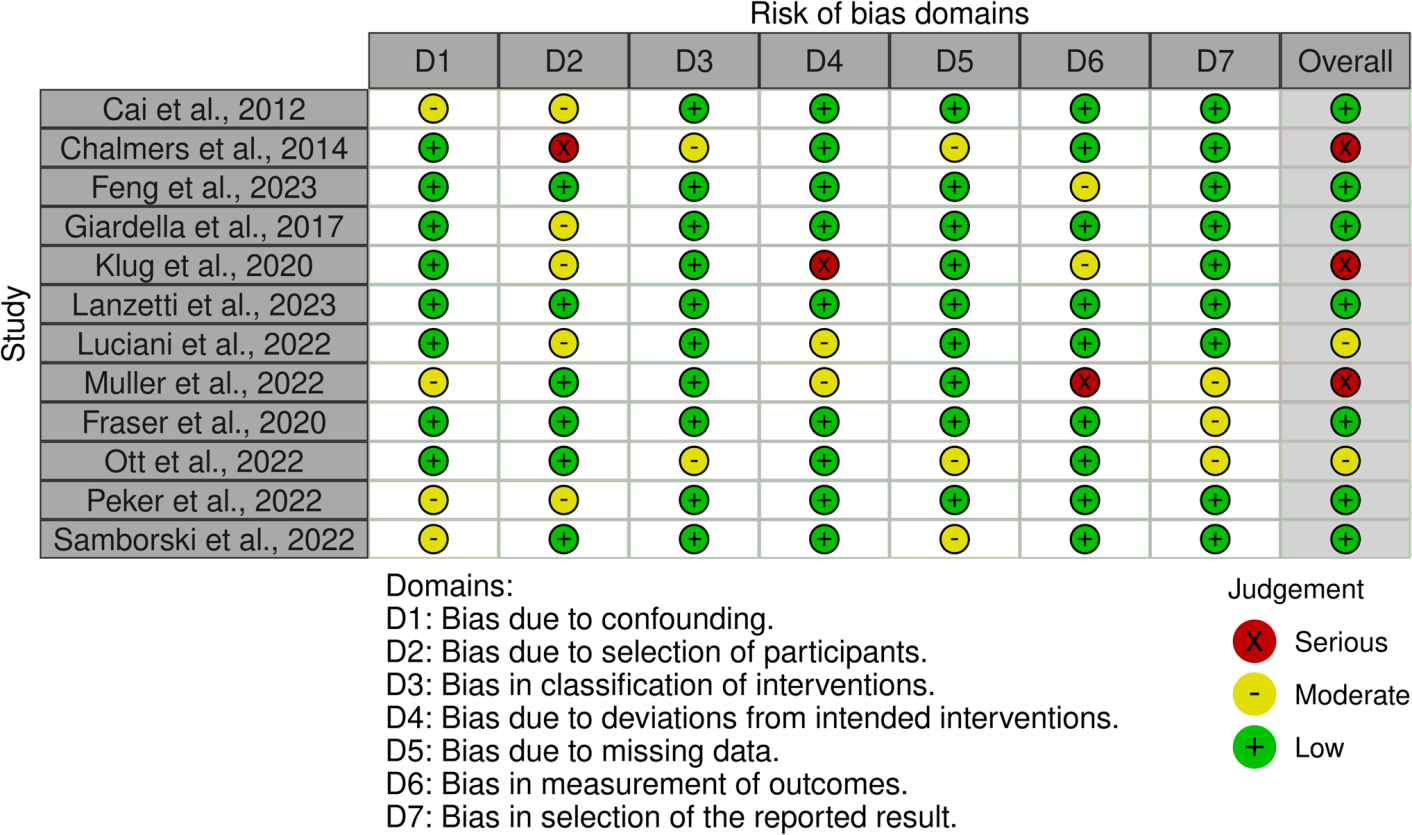
The ROBINS-I of non-RCTs

The ROBINS-I was applied to investigate the risk of bias for all articles included in the present study, as only non-RCTs were reviewed. No major concerns were present in most studies, and only a few had minor concerns, resulting in a predominantly low-risk rating in this domain. The lack of participant data for a defined follow-up period led to a serious risk of selection bias in one of the included studies. Other serious risks of bias were identified in one study because of deviations from the planned intervention and in another because of concerns about outcome measurement. Bias during the intervention was mostly low in the remaining studies, as were the domains assessed for risk of bias after the intervention, due to missing data and the reported outcome selection. Given the broadly acceptable quality of the included studies, the overall risk of bias was low to moderate (Fig. 3).

### Study characteristics and results of individual studies

Data from 878 procedures were collected. 72% (631 of 878 patients) were women. The mean length of the follow-up was 33.8 ± 14.8 months. The mean age of the patients was 73.4 ± 4.6 years. The generalities and demographics of the included studies are shown in Table 1. All pooled data from complications for the analysis of each procedure are reported alphabetically in Appendix B.

**Table 1:** Generalities and patient baseline of the included studies

Author	Journal name	Design	Groups	Follow-up (*month*)	Patients (*n*)	Mean age	Women (*n*)
Cai et al., 2012 [50]	*Orthopedics*	Prospective	ORIF	24.3	13	71.1	11
			HA	24.3	19	72.4	16
Chalmers et al., 2014 [51]	*J Shoulder Elbow Surg*	Retrospective	ORIF	36.0	9	71.0	7
			HA	58.8	9	72.0	7
			rTSA	14.4	9	77.0	7
Feng et al., 2023 [52]	*Heliyon*	Retrospective	ORIF	37.4	36	71.3	29
			HA	56.1	10	73.5	7
Fraser et al., 2020 [37]	*J Bone Joint Surg Am*	Prospective	ORIF	24.0	60	74.7	52
			rTSA	24.0	64	75.7	59
Giardella et al., 2017 [38]	*Muscles Ligaments Tendons J*	Retrospective	ORIF	40.0	23	72.1	16
			rTSA	24.0	21	77.2	18
Klug et al., 2020 [39]	*J Shoulder Elbow Surg*	Retrospective	ORIF	49.0	30	72.5	5
			rTSA	38.0	30	73.9	5
Lanzetti et al., 2023 [53]	*Clin Orthop Relat Res*	Prospective	ORIF	53.0	66	73.0	29
			rTSA	53.0	72	76.0	40
Luciani et al., 2022 [54]	*Musculoskelet Surg*	Retrospective	ORIF	40.0	26	73.0	25
			rTSA	33.4	22	75.5	20
Muller et al., 2022 [55]	*Arch Orthop Trauma Surg*	Retrospective	ORIF	52.5	63	71.4	46
			rTSA	33.5	40	76.8	34
Ott et al., 2022 [56]	*Ota Int*	Retrospective	ORIF		90	81.9	76
			rTSA		71	82.3	56
Peker et al., 2022 [57]	*J Pak Med Assoc*	Retrospective	ORIF	18.3	30	60.0	14
			HA	18.7	18	67.3	11
Samborski et al., 2022 [58]	*J Shoulder Elbow Surg*	Retrospective	ORIF	12.0	23	67.1	18
			rTSA	12.0	24	77.3	23

*ORIF* open reduction and internal fixation; *rTSA* reverse total shoulder arthroplasty; *HA* hemiarthroplasty

### Baseline comparability

Comparability was found between groups regarding mean age, men:women ratio, length of follow-up, and time elapsed from injury to the index procedure (Table 2).

**Table 2:** Baseline comparability (FU: follow-up; ORIF: open reduction and internal fixation; rTSA: reverse total arthroplasty)

Endpoint	Hemiarthroplasty (n = 56)	ORIF (n = 469)	rTSA (n = 353)	*P*
Last FU (*month*)	39.5 ± 20.9	35.1 ± 13.9	29.0 ± 13.4	0.3
Mean age	71.3 ± 2.7	71.6 ± 5.0	76.9 ± 2.3	0.1
Women (%)	73% (41 of 56)	70% (328 of 469)	76% (262 of 353)	0.5
Time to procedure (*days*)	11.4 ± 8.0	5.1 ± 3.0	5.0 ± 1.8	0.09

### Synthesis of results

**Fig. 4:**
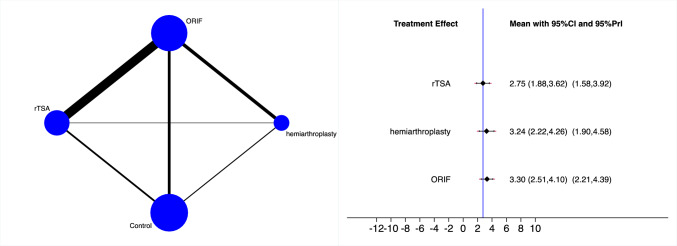
Edge and interval plot of the comparison: complication

rTSA demonstrated the lowest rate of complications, followed by hemiarthroplasty and ORIF (Fig. 4). The equation for global linearity found no statistically significant inconsistency (P_Wald_ = 0.7).

**Fig. 5:**
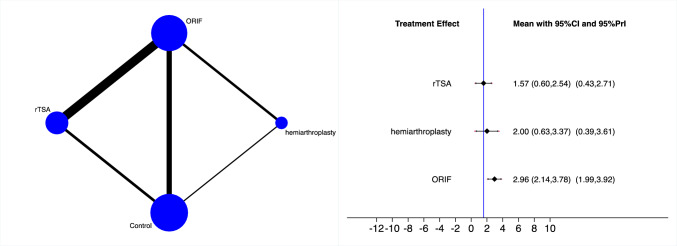
Edge and interval plot of the comparison: revision

rTSA demonstrated the lowest rate of revision, followed by hemiarthroplasty and ORIF (Fig. 5). The equation for global linearity found no statistically significant inconsistency (P_Wald_ = 0.7).

## Discussion

According to the main findings of the present study, for patients above 60 with PHF in whom surgery was performed, rTSA was associated with a lower rate of complications and revision compared to ORIF and HA. These results contribute to the ongoing clinical discussion regarding the optimal surgical strategy in this population.

Osteosynthesis with preservation of the humeral head is recommended when adequate reduction can be obtained [31, 35]. However, avascular necrosis might occur, given the terminal vascularisation of the humeral head. Moreover, given the relatively high complication rate, alternative surgical options, such as arthroplasty and non-operative management, have been advocated [32–34]. ORIF performs better than hemiarthroplasty in less comminuted fractures, leading to better outcomes and range of motion with similar complication rates, improving the bending and torsion resistance [50, 59]. However, in comminuted PHF, HA was associated with lower pain on the VAS than ORIF [57, 60, 61]. Feng et al. [52] compared ORIF in adults (n = 24) versus ORIF in elderly patients (n = 36) versus HA (n = 10) in patients with locked fracture-dislocations of the proximal humerus. For patients younger than 60 years, ORIF might be optimal, whereas for patients older than 60 years, both ORIF and HA provided similar results [52]. However, ORIF was associated with a higher rate of complications [52].

rTSA might promote greater joint function than HA and ORIF [2, 38]. rTSA results in greater function than ORIF in bifocal extra-articular and intra-articular PHFs in 124 patients above 60 [37]. These findings are supported by previous clinical investigations, which recommended rTSA in older adults with dislocated PHFs, especially for its lower complication rate and revision [39, 54, 55, 58]. The superiority of rTSA to ORIF and HA was also confirmed in previous meta-analyses [37, 42, 43, 62]. However, there are no clear indications for rTSA [40, 41]. rTSA might be performed in non-reconstructable head-splitting fractures, fractures devoid of all soft tissue attachments, severe valgus-impacted fractures with disruption of the medial periosteal hinge, and displaced multi-fragmentary fractures with delayed presentation (more than four weeks from injury) [63–65]. HA was introduced to overcome the limitations of ORIF in patients with complex PHFs who required humeral head excision [28, 36]. However, HA is becoming increasingly less common, given the introduction of innovative locking plates and the good outcomes in rTSA [28, 66, 67]. HA is limited to patients with comminuted head fractures with intra-articular involvement and intact glenoid [28, 68, 69]. Instability, rotator cuff dysfunction, and tuberosity non-union leading to functional deficits represent possible complications of HA. In addition, the results of the present study indicate that HA is associated with a greater rate of complications and revision compared to rTSA. On the other hand, in patients with good shoulder function without advanced osteoarthritis and glenoid damage, HA spares the glenoid instrumentation, shortens surgical time, and reduces surgery-related costs.

Previous evidence is conflicting. Gallinet et al. [42] conducted a systematic review and meta-analysis of 22 studies comparing rTSA and HA in elderly patients with displaced proximal humeral fractures. While rTSA demonstrated superior functional outcomes in forward flexion and abduction, HA was associated with a lower complication rate [42]. The reoperation rate was comparable between groups, whereas the revision rate (implant exchange) was higher after HA [42]. Suroto et al. [43] performed a meta-analysis of six comparative studies evaluating rTSA versus ORIF for three- and four-part fractures. Their results have shown that rTSA was associated with improved forward flexion and constant scores but also a higher complication rate compared to ORIF during early follow-up [43]. In contrast, the present analysis suggests that rTSA is associated with a lower rate of complications and revisions than ORIF and HA, particularly in older adults. In addition to these surgical outcomes, rTSA also tended to be associated with improved functional recovery, as reflected by higher Constant and ASES scores and lower VAS pain scores in several studies. A formal statistical comparison was not feasible given the inconsistent reporting and missing measures of dispersion. The discrepancy with previous meta-analyses may be attributable to differences in inclusion criteria, temporal shifts in surgical practice, and the incorporation of more recent evidence in the present study. Furthermore, applying a Bayesian network model instead of conventional pairwise comparisons may have contributed to the variation in findings by integrating direct and indirect evidence.

This study has several limitations, including the limited number of included studies, relative sample size, surgical techniques and type of implants, and the retrospective nature of most studies. The length of the follow-up was variable. Although several studies reported data on the ROM, the reporting was inconsistent across studies regarding completeness, measurement methods (e.g., active vs. passive ROM), and availability of statistical dispersion (e.g., standard deviations). In many cases, only partial ROM data were reported, but not all movements (flexion, abduction, external rotation) were consistently assessed across all surgical groups. Given these limitations, comparing the ROM was not possible in a network modality. Given the variability in the definition of complications and their reporting across studies, a standardised categorisation with relative risk calculation and network comparison was not feasible without introducing bias. While some studies did report revision procedures in detail, others provided only general information, and definitions varied considerably. In addition, the complication rate was mainly low, which impaired the Bayesian model analysis in detecting a ranking of interventions. Therefore, a structured comparison of revision types was not performed. In some studies, the short follow-up might jeopardise the detection of long-term complications, especially regarding avascular necrosis or loosening of the prosthesis. Most studies did not specify the type of fractures or did not refer to the international classification; therefore, additional subgroup analyses according to the fracture classification were not possible. The present Bayesian network meta-analysis did not consider hardware removal. Hardware removal might be necessary for patients with stiffness, screw migration, or mechanical impingement, which represent surgical complications. On the other hand, hardware removal might follow patients’ wishes and the healthcare system or health insurance indications, which is not a surgical complication. However, since most studies did not report information on the surgical indication, hardware removal was not considered for analysis. Despite this limitation, ORIF showed the highest complication rate, irrespective of hardware removal. Many included studies did not report clinical data or reported patient-reported outcome measures (PROMs) without any statistical measures of dispersion (e.g. standard deviation); therefore, given the lack of exhaustive data available for inclusion, a comparison of the clinical outcome between the three techniques could not be conducted in a network fashion. Future studies should overcome this limitation. The rTSA group has a shorter follow-up length than the other groups. Although these data were not statistically significant, they might induce an underestimation of the rate of complications of rTSA. In addition, one article did not report information on the length of the follow-up. Given the small number of included studies, the power to detect asymmetry of Egger’s test and funnel plots is limited. Additional studies should evaluate patients according to internationally accepted classifications to refine indications and outcomes and provide longer follow-ups. Future studies should also assess which patients are suitable for conservative management or surgical treatment, integrating the advantage of current developments in artificial intelligence tools into patient care pathways [70].

## Conclusion

In patients above 60 with PHF, rTSA was associated with a lower rate of complications and revision compared to ORIF and HA. Future investigations are required to validate these results on a larger scale and elaborate internationally accepted guidelines and therapeutic algorithms for managing PHF in this age group.

## Supplementary Information

Below is the link to the electronic supplementary material.

Supplementary file1 (DOCX 17 KB)

Supplementary file2 (DOCX 20 KB)

## Data Availability

The datasets generated during and/or analysed during the current study are available throughout the manuscript.
