# Supplementary material for: Reverse arthroplasty compared to hemiarthroplasty and open reduction and internal fixation for displaced proximal humerus fracture in patients above 60: a Bayesian network meta-analysis
Source: Arch Orthop Trauma Surg. 2025 Oct 18;145(1):481. doi: 10.1007/s00402-025-06067-5 (PMC12535496; doi:10.1007/s00402-025-06067-5)
Supplement: Supplementary file 1 — Supplementary file1 (DOCX 17 KB) [file 402_2025_6067_MOESM1_ESM.docx]

**Systematic literature search for:**

**Reverse arthroplasty compared to hemiarthroplasty and open reduction and internal fixation of displaced shoulder fracture in the elderlies: a Bayesian network meta-analysis**

**Concept 1: Disease**

**Keywords:**

shoulder

displaced shoulder fracture

fracture dislocation

**MeSH:**

"Shoulder"[Mesh]

"Shoulder Fractures"[Mesh]

"Fracture Dislocation"[Mesh]

**Concept 2: Therapy**

**Keywords:**

arthroplasty, replacement

shoulder arthroplasty

hemiarthroplasty

reverse arthroplasty

reverse shoulder arthroplasty

reverse total shoulder arthroplasty

open reduction

open fracture reduction

fracture fixation

internal fracture fixation

**MeSH:**

"Shoulder/surgery"[Mesh]

"Arthroplasty, Replacement, Shoulder"[Mesh]

"Hemiarthroplasty"[Mesh]

"Open Fracture Reduction"[Mesh]

"Fracture Fixation"[Mesh]

"Fracture Fixation, Internal"[Mesh]

**Concept 3: Further points of interest**

**Keywords:**

elderlies

aged

**MeSH:**

"Aged"[Mesh]

**Concept 1:**

"Shoulder"[Mesh] OR "Shoulder Fractures"[Mesh] OR "Fracture Dislocation"[Mesh] OR shoulder OR displaced shoulder fracture OR fracture dislocation

**AND**

**Concept 2:**

"Shoulder/surgery"[Mesh] OR "Arthroplasty, Replacement, Shoulder"[Mesh] OR "Hemiarthroplasty"[Mesh] OR "Open Fracture Reduction"[Mesh] OR "Fracture Fixation"[Mesh] OR "Fracture Fixation, Internal"[Mesh] OR shoulder arthroplasty OR hemiarthroplasty OR reverse shoulder arthroplasty OR reverse total shoulder arthroplasty OR open fracture reduction OR fracture fixation OR internal fracture fixation

**AND**

**Concept 3:**

"Aged"[Mesh] OR aged OR elderlies

**Summary of the Search**

(("Shoulder"[Mesh] OR "Shoulder Fractures"[Mesh] OR "Fracture Dislocation"[Mesh] OR shoulder OR displaced shoulder fracture OR fracture dislocation) AND ("Shoulder/surgery"[Mesh] OR "Arthroplasty, Replacement, Shoulder"[Mesh] OR "Hemiarthroplasty"[Mesh] OR "Open Fracture Reduction"[Mesh] OR "Fracture Fixation"[Mesh] OR "Fracture Fixation, Internal"[Mesh] OR shoulder arthroplasty OR hemiarthroplasty OR reverse shoulder arthroplasty OR reverse total shoulder arthroplasty OR open fracture reduction OR fracture fixation OR internal fracture fixation)) AND ("Aged"[Mesh] OR aged OR elderlies)
