# Supplementary material for: Reverse arthroplasty compared to hemiarthroplasty and open reduction and internal fixation for displaced proximal humerus fracture in patients above 60: a Bayesian network meta-analysis
Source: Arch Orthop Trauma Surg. 2025 Oct 18;145(1):481. doi: 10.1007/s00402-025-06067-5 (PMC12535496; doi:10.1007/s00402-025-06067-5)
Supplement: Supplementary file 2 — Supplementary file2 (DOCX 20 KB) [file 402_2025_6067_MOESM2_ESM.docx]

| **Type of complications** | **Hemiarthroplasty** | **ORIF** | **rTSA** |
| --- | --- | --- | --- |
| Adhesive capsulitis | 0 | 4 | 0 |
| Arthrofibrosis | 1 | 1 | 0 |
| Axillary nerve injuries | 0 | 0 | 1 |
| Complete avascular necrosis of the humeral head | 0 | 18 | 0 |
| Complex regional pain syndrome | 0 | 0 | 1 |
| Cranialization of the greater tuberosity | 0 | 0 | 0 |
| Death | 1 | 4 | 5 |
| Delirium | 0 | 0 | 4 |
| Dislocation | 1 | 0 | 2 |
| Dislocation of the fragments | 0 | 3 | 0 |
| Fixation failures | 0 | 15 | 0 |
| Fractures overall | 0 | 1 | 0 |
| Haematoma post operative | 0 | 4 | 1 |
| Heterotopic ossification | 0 | 1 | 0 |
| Implant Problems | 0 | 9 | 0 |
| Infection | 1 | 1 | 5 |
| Inferior scapular notching | 0 | 0 | 7 |
| Instability | 0 | 0 | 1 |
| Lysis of greater tuberosity | 0 | 0 | 1 |
| Lysis of lesser tuberosity | 0 | 0 | 1 |
| Malunion | 6 | 7 | 0 |
| Malunion of greater tuberosity | 0 | 2 | 5 |
| Metal hypersensitivity | 0 | 0 | 1 |
| Necrosis | 0 | 8 | 0 |
| Nerve injury | 0 | 0 | 4 |
| Non-union | 0 | 3 | 0 |
| Partial avascular necrosis of the humeral head | 0 | 4 | 0 |
| Partial reabsorption of greater tuberosity | 0 | 4 | 6 |
| Partial reabsorption of lesser tuberosity | 0 | 1 | 0 |
| Perioperative glenoid fracture | 0 | 0 | 1 |
| Periprosthetic Fracture | 0 | 0 | 3 |
| Posttraumatic shoulder stiffness | 0 | 6 | 0 |
| Prothesis loosening | 1 | 0 | 0 |
| Rotator cuff ruptures | 0 | 1 | 0 |
| Scapular spur | 0 | 0 | 1 |
| Screw cut-out | 0 | 16 | 0 |
| Subacromial plate impingement | 0 | 2 | 0 |
| Subluxation | 2 | 0 | 0 |
| Ulnar neuritis at the cubital tunnel | 1 | 0 | 0 |
| Unspecified | 14 | 98 | 41 |
